# The impact of a PERMA model-based multi-component positive psychology group intervention on nurse second victims

**DOI:** 10.3389/fpsyg.2026.1941464

**Published:** 2026-09-25

**Authors:** Hui Chen, Li Wang, Haiyan Liu

**Affiliations:** Linyi People's Hospital, Linyi, China

**Keywords:** group intervention, nurse second victims, PERMA model, positive psychology, psychological capital, support resource efficacy

## Abstract

**Objective:**

This study aimed to explore the application effects of a multi-component positive psychological group intervention grounded in the PERMA model on anxiety, psychological capital, and support resource efficacy among nurse second victims.

**Methods:**

This study was a single-center randomized controlled trial. A total of 86 nurses who had experienced adverse events and met the inclusion and exclusion criteria were recruited via convenience sampling from a Class A tertiary comprehensive hospital in Shandong Province between December 2025 and February 2026. After recruitment, participants were randomly assigned to the control group (*n* = 43) and the intervention group (*n* = 43) using a computer-generated random sequence concealed in sequentially numbered opaque envelopes. Due to sample attrition during the intervention, 42 nurses in the intervention group and 41 nurses in the control group ultimately completed the study. The intervention group received a 6-week multi-component positive psychology group intervention based on the PERMA model, whereas the control group received only routine hospital support. The Generalized Anxiety Disorder-7 (GAD-7), the Psychological Capital Questionnaire-R (PCQ-R), and the Second Victim Experience and Support Tool (SVEST) were adopted to evaluate outcomes before intervention, immediately after the intervention, and at the 3-month follow-up (T3). Data were analyzed using independent sample *t*-tests, repeated measures ANOVA, and chi-square tests.

**Results:**

Immediately after the intervention and at the 3-month follow-up, the intervention group exhibited significantly lower anxiety scores and second victim experience and support scores, as well as remarkably higher psychological capital scores compared with the control group (all *p* < 0.05).

**Conclusion:**

The multi-component positive psychology group intervention based on the PERMA model can effectively alleviate anxiety among nurse second victims and enhance their internal psychological resources and capacity to utilize external support.

## 1 Introduction

Nursing adverse events refer to unplanned abnormal incidents inconsistent with expected nursing objectives that occur during nursing practice (Wang et al., 2025), serving as one of the key indicators for the quality of nursing services. The term “second victim” (SV) describes medical workers who suffer physical and psychological trauma after directly or indirectly being involved in unexpected medical errors or adverse events (Choi et al., 2022). As frontline practitioners delivering clinical nursing care, nurses are more vulnerable to patient safety incidents and may become second victims due to understaffing, excessive workloads, and high occupational risks (Catalán et al., 2024). Such adverse experiences further trigger negative emotions, including anxiety and depression, accompanied by reduced work enthusiasm, weakened professional identity and elevated turnover intention (Hakamy et al., 2025; Xie et al., 2026). Research indicates (Yang and Zhang, 2024) that higher levels of psychological capital and effective support can substantially mitigate the negative experiences of nurse second victims and facilitate their coping with the detrimental consequences following adverse patient events. Accordingly, mitigating psychological stress, boosting psychological capital and optimizing support availability among nurse second victims have emerged as urgent research priorities.

Positive psychology intervention is a therapeutic approach rooted in positive psychology theories and delivered through positive practical activities, which exerts critical effects on enhancing individuals’ well-being, psychological resilience, and positive psychological traits (Arslan et al., 2025). Previous research has demonstrated (Peng and Xu, 2023) that group interventions based on positive psychology can significantly reduce nurses’ occupational burnout, strengthen their psychological resilience, and relieve negative emotions such as anxiety and depression. However, existing research on the psychological wellbeing and support status of nurse second victims has mainly comprised cross-sectional studies and lacks systematic positive psychology intervention programs.

The PERMA theoretical model, proposed by Martin Seligman, is an influential multidimensional well-being framework within positive psychology. It encompasses five dimensions: positive emotion, engagement, relationships, meaning, and accomplishment (Kovich et al., 2023). Among these, the positive emotion and engagement dimensions can effectively alleviate negative emotions such as anxiety and depression by fostering positive emotions and promoting work engagement; the accomplishment and meaning dimensions can enhance self-efficacy, cultivate a positive and optimistic mindset, and boost psychological capital by setting phased goals and guiding individuals to reframe the perceived value of adverse life events; the relationships dimension can improve social connectedness and enhance the ability to utilize external support through family support and peer assistance. Prior studies have shown that multi-component positive psychology group interventions based on the PERMA model can effectively mitigate negative emotions and improve psychological wellbeing among patients with chronic diseases (Luo et al., 2025). However, the effectiveness of this intervention model for nurses experiencing secondary victimization remains unconfirmed. Therefore, this study aims to evaluate the effects of a multi-component positive psychology group intervention based on the PERMA model on nurses experiencing secondary victimization.

In summary, we hypothesize a significant Group × Time interaction. Relative to the conventional control group, nurse second victims in the intervention group will exhibit significantly reduced anxiety, alongside sustained improvements in their internal psychological resources and the ability to utilize external support; furthermore, these effects will be statistically significant both immediately after the intervention and at the 3-month follow-up.

## 2 Materials and methods

### 2.1 Study design

This study is a single-center, randomized controlled trial designed to assess the effectiveness of a 6-week, multi-component positive psychology group intervention based on the PERMA model for nurse second victims. The study protocol was reviewed and approved by the hospital’s ethics committee (Approval No: 202511-H-019). All participants provided informed consent. After recruitment, eligible participants were randomly assigned to the control group and the intervention group using a computer-generated random sequence concealed in sequentially numbered opaque envelopes; the allocation sequence was generated by a statistician independent of intervention delivery, and group assignment was revealed only after baseline assessments were completed. In addition, due to the nature of the intervention, only outcome assessors and data collectors were blinded in this trial.

### 2.2 Settings and participants

Study participants consisted of 86 nurse second victims recruited from a Grade III Class A general hospital in Shandong Province (Figure 1). Inclusion criteria: (1) Registered nurses with valid practicing certificates in active employment; (2) Clinical nurses who had personally experienced nursing adverse events within the preceding 2 months; (3) A total score ≥ 10 on the Generalized Anxiety Scale; (4) Basic proficiency in smartphones and WeChat operation; (5) Voluntary participation with signed informed consent. Exclusion criteria: (1) Locum nurses, student nurses, probationary nurses and nurses undergoing standardized residency training; (2) Nurses planning extended leave or resignation during the research period; (3) Nurses receiving systematic psychological counseling or pharmacotherapy for mental health disorders; (4) Participants concurrently enrolled in other psychological intervention studies.

**Figure 1:**
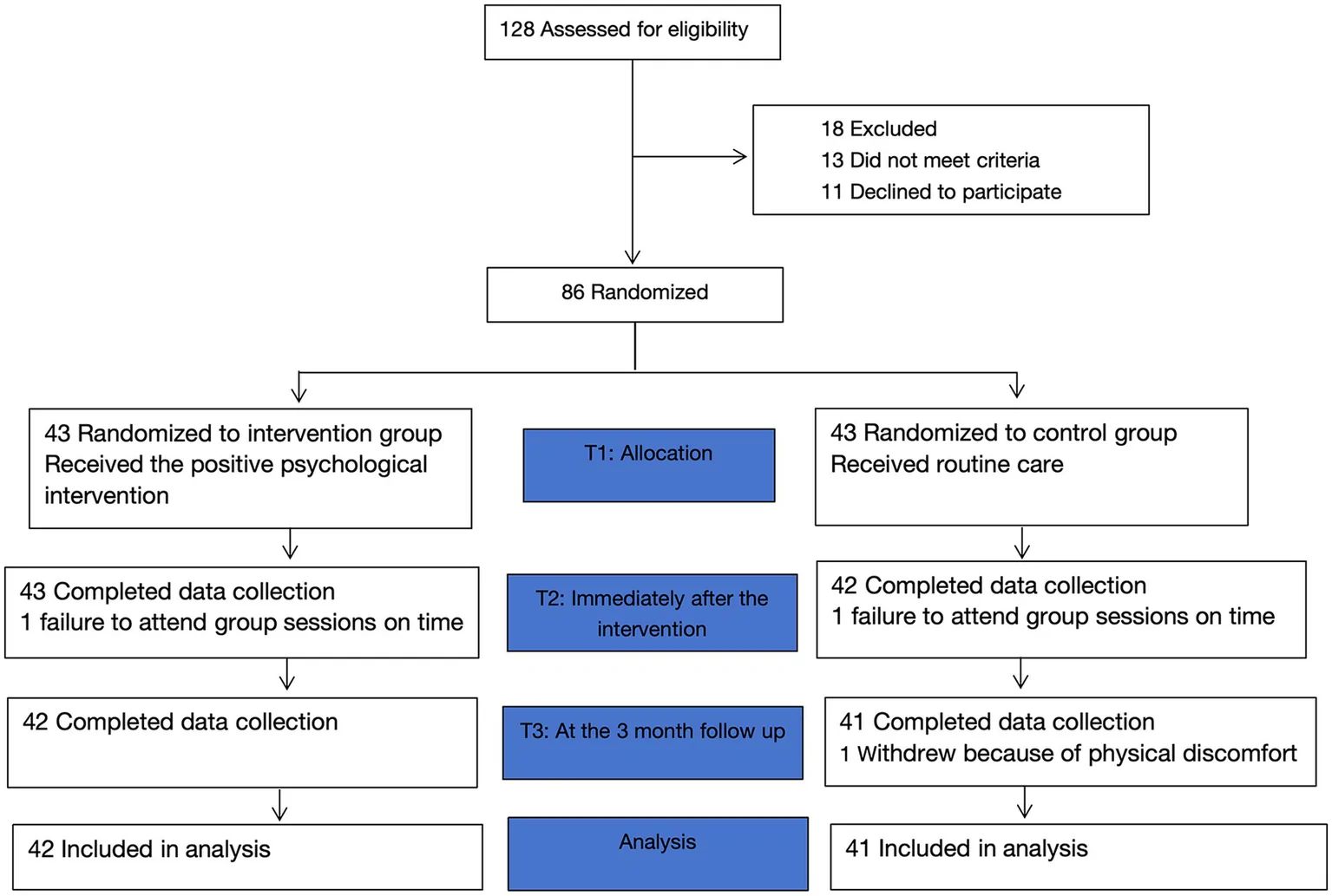
Flow chart of participant’s enrollment.

### 2.3 Control group

Participants in the control group received general psychological support from the head nurse and other hospital staff, and also attended the hospital’s mental health education programs.

### 2.4 Intervention group

On the basis of the routine support provided to the control group, participants received a multi-component positive psychology group intervention grounded in the PERMA model. Research team composition: The research team comprised seven members: one psychology expert responsible for formulating the intervention protocol and providing professional guidance; one nursing administrator acting as coordinator to arrange learning activities and oversee overall coordination; one psychological counselor and two well-being instructors in charge of routine group training and protocol revision; two postgraduate nursing students responsible for data collection and statistical analysis.

Development of the Intervention Program: Guided by the PERMA theoretical framework, informed by a literature review, and considering the psychological characteristics of nurse second victims, we developed an initial multi-component group intervention program centered on the themes of positive emotions, positive engagement, positive relationships, positive meaning, positive achievements, and goals. The program incorporated activities such as emotion management, mindfulness training, and social support mapping. Following program evaluation and revision through expert meetings, six nurse second victims were recruited for a pilot study. We collected their feedback, made appropriate adjustments to the intervention measures, and finalized the intervention program.

Implementation of the Intervention Program: The intervention adopted a hybrid online–offline delivery model. Offline sessions were delivered weekly as group counseling, lasting 1 h per session over 6 weeks. Each offline session was facilitated by one nursing administrator, one psychological counsellor and two well-being instructors. Session activities included emotional regulation training, education on flow-state experiences, doctor-patient communication skill training, character strength exploration, etc. One week before each group activity, the team coordinator notified all participants of the training schedule and coordinated nursing work shifts. The online part, serving as an extension and supplement to offline group activities, was delivered via the “NurseMind” mini-program. After each weekly offline class, positive psychology content relevant to offline topics was posted on the mini-program. The platform hosted a Mindfulness-Based Stress Reduction course and online counseling services. Participants could access supporting materials repeatedly at their own pace for self-directed improvement. A multi-component positive psychology group intervention program based on the PERMA model is presented in Table 1.

**Table 1:** Multi-component positive psychology group intervention program based on the PERMA model.

Unit	Theme	Objectives	Activities
I	Positive Emotions	1. Establish the group, clarify group norms and objectives, and build trusting relationships among members 2. Recognize and accept emotions; foster positive cognition and cultivate positive emotions	1. Lectures covering “positive psychology” and “stress relief” 2. Emotional management training: equip nurses with skills to identify emotions in themselves and others, and master strategies for regulating positive emotions 3. On-site practice using the emotional first-aid toolkit: simulate stressful scenarios and apply the “5–4 - 3 - 2 - 1” sensory grounding technique to rapidly relieve anxiety 4. Brief mindfulness breathing exercise
II	Positive Engagement	1. Identify a sense of occupational meaning and mission, and develop a positive attitude toward setback	1. Learn theories of flow-state experiences 2. Participate in interest-based activities to experience the pleasure of engagement 3. Share personal experiences of full engagement 4. Brief mindfulness breathing exercise
III	Positive Relationships	1. Identify personal communication styles and learn positive communication patterns 2. Perceive social support, cultivate gratitude and build harmonious interpersonal relationships.	1. Doctor-patient communication skills training, including active listening, nonverbal communication, and emotional soothing techniques 2. Conduct the Positive Relationship Tree activity 3. Guide participants to offer positive and constructive feedback to others 4. Design a “social support map” to visualize individual support resources (colleagues, family, professional psychological counseling, etc.)
IV	Positive Meaning	1. Further explore self-identity and clarify personal life value and meaning 2. Cultivate self-confidence and a sense of occupational accomplishment	1. Introduce knowledge about adverse events and second victims; help participants recognize their identity as victims of such events and fully utilize available support resources 2. Guided reflective writing to reframe narratives of adverse events, focusing on lessons learned rather than failures. Participants are required to identify at least three positive transformations brought by the incidents (e.g., improved skills, enhanced awareness of teamwork) 3. Strength Exploration Activity: review successful cases throughout their professional career 4. Brief mindfulness breathing exercise
V	Positive Accomplishment and Goal Setting	1. Convey the core notion of positive goal pursuit: value lies not only in goal achievement, but also in the process of selecting and pursuing goals	1. Introduce methods to cultivate positive accomplishment 2. Share personally fulfilling work experiences, recognize individual achievements, set short-term goals, and sustain efforts toward these goals 3. Discuss future career prospects to strengthen participants’ confidence and hope for their work 4. Brief mindfulness breathing exercise
VI	Summary	1. Cultivate gratitude in daily life and enhance nurses’ subjective well-being	1. Daily practice of the three good things exercise 2. Collect feedback regarding group intervention effects; transfer knowledge and skills acquired in the group to daily life and work

### 2.5 Data collection and outcome measures

Data collection and data cleaning were conducted by the researcher and two nursing postgraduates. At baseline before the intervention (T1), enrolled nurses completed the general information questionnaire, GAD-7, PCQ-R, and the SVEST scale. Follow-up assessments using the same scales were carried out immediately after intervention (T2, after the final group counseling session) and at 3 months post-intervention (T3). Electronic questionnaires were created on an online survey platform, with standardized instructions provided to all participants. Functions such as mandatory response for missing items and limited submission attempts were configured to ensure complete questionnaire responses. Following informed consent, participants completed the questionnaires anonymously. Two nursing postgraduates screened all returned questionnaires, and excluded invalid questionnaires.

The Generalized Anxiety Disorder Scale (GAD-7) was used to assess anxiety levels among nurse second victims. Originally developed by Spitzer et al. (2006), the scale was translated into Chinese and validated by He et al. (2010). It contains 7 items, with total scores ranging from 0 to 21. Response options (“not at all”, “several days”, “more than half the days”, and “almost every day”) are assigned scores of 0, 1, 2, and 3, respectively. A higher total score indicates more severe anxiety symptoms. The Cronbach’s α coefficient in the present sample was 0.898.

The Psychological Capital Questionnaire-R (PCQ-R) was developed by Luthans et al. (2004) and subsequently adapted and validated for Chinese populations by Luo and He (2010). It comprises 20 items distributed across 4 dimensions. Response options (“Strongly Disagree,” “Disagree,” “Somewhat Disagree,” “Somewhat Agree,” “Agree,” and “Strongly Agree”) are scored 1, 2, 3, 4, 5, and 6, respectively. A higher score indicates greater psychological capital among nurses, and the Cronbach’s α coefficient is 0.923. In the present study, the Cronbach’s α coefficient for the total scale was 0.87, and the McDonald’s ω coefficient was 0.88.

The Second Victim Experience and Support Tool (SVEST), translated and revised by Chen (2020), was adopted in this study. This scale includes 24 items across 6 dimensions, among which the “Support” dimension adopts reverse scoring. Total scores range from 24 to 120; a higher score indicates lower perceived efficacy of support resources for nurse second victims. The Cronbach’s α coefficient for the current sample was 0.824.

### 2.6 Statistical analysis

Statistical analyses were performed using SPSS 27.0. Categorical data were described as frequencies and percentages. Between-group comparisons were conducted using the Pearson *χ^2^* test; Fisher’s exact test was adopted when theoretical frequencies failed to meet the prerequisites for the *χ^2^* test. The Shapiro–Wilk test was used to assess the normality of continuous data. Normally distributed continuous variables were presented as mean ± standard deviation, with 95% confidence intervals for the mean, and compared between groups using the independent-samples *t*-test. Non-normally distributed continuous variables were expressed as median (interquartile range) [M(P25, P75)], and between-group comparisons were performed with the Mann–Whitney *U* test. A 2-group × 3-time-point mixed-design repeated-measures analysis of variance (ANOVA) was applied to examine changes in GAD-7, PCQ-R, and SVEST scores at T1, T2, and T3, testing the main effects of time and group as well as the time-by-group interaction effect. Mauchly’s sphericity test was used to verify the sphericity assumption. The Greenhouse–Geisser correction was applied to adjust degrees of freedom if the sphericity assumption was violated. For repeated-measures ANOVA, the *F* statistic, *p*-value, and partial η^2^ were reported as the effect size. Simple effect analysis was performed when the time-by-group interaction reached statistical significance, to compare between-group differences at each time point and within-group differences across time points. The Bonferroni method was used to correct for multiple comparisons. All hypothesis tests were two-tailed, and a *p*-value less than 0.05 was considered statistically significant.

### 2.7 Sample size

The sample size was calculated using the formula for comparing means between two independent groups: n1 = n2 = 2[(μ_α_ + μ_β_)σ/δ]^2^. A two-sided significance level *α* = 0.05, *β* = 0.10 were specified. Based on a prior comparable study (Chang et al., 2025), the mean difference δ = 3.30 and standard deviation σ = 3.89. The calculation yielded a minimum required sample of 23 participants per group. Accounting for a 10–15% dropout rate and practical feasibility, a minimum total sample of 52 participants was required. Ultimately, 86 participants were recruited into this study, with 43 allocated to the intervention group and 43 to the control groups.

## 3 Results

### 3.1 Comparison of baseline characteristics between the two groups of nurse second victims

During the research, two participants (one from each group) were lost to follow-up owing to missed group sessions, and one participant withdrew because of physical discomfort. Ultimately, 83 participants completed the research, including 42 nurses in the intervention group and 41 nurses in the control group. No statistically significant differences were observed for baseline characteristics between the two groups (*p* > 0.05), indicating that the groups were comparable. See Table 2.

**Table 2:** Comparison of baseline characteristics between the two groups of nurse second victims [*n* (percentage, %)].

Item	Category	Intervention Group (n = 42)	Control Group (n = 41)	χ^2^	*p*
Age	20–29 years	8(19.05)	3(7.32)	3.283	0.350
	30–39 years	23(54.76)	22(53.66)		
	40–49 years	10(23.81)	15(36.59)		
	≥50 years	1(2.38)	1(2.44)		
First degree	Secondary vocational school	9(21.43)	8(19.51)	0.647	0.723
	Junior College	20(47.62)	23(56.10)		
	Bachelor’s degree or above	13(30.95)	10(24.39)		
Highest education	Junior college	0(0.00)	1(2.44)	1.037	0.309
	Bachelor’s degree or above	42(100.00)	40(97.56)		
Marital Status	Unmarried	6(14.29)	2(4.88)	3.205	0.201
	Married	35(83.33)	39(95.12)		
	Divorced	1(2.38)	0(0.00)		
Professional Title	Nurse	3(7.14)	1(2.44)	2.681	0.444
	Senior nurse	7(16.67)	5(12.20)		
	Senior nurse	24(57.14)	30(73.17)		
	Associate chief nurse	8(19.05)	5(12.20)		
Working department	Internal medicine	11(26.19)	6(14.63)	13.485	0.061
	Surgery	14(33.33)	6(14.63)		
	Emergency department	1(2.38)	2(4.88)		
	ICU	2(4.76)	3(7.32)		
	Operating room	1(2.38)	0(0.00)		
	Pediatrics	6(14.29)	5(12.20)		
	Obstetrics and gynecology	4(9.52)	16(39.02)		
	Others	3(7.14)	3(7.32)		
Employment status	Public institution staffing	12(28.57)	14(34.15)	0.300	0.584
	Contract staff	30(71.43)	27(65.85)		
Monthly personal income	≤5,000 CNY	2(4.76)	1(2.44)	3.855	0.278
	5,000–10,000 CNY	32(76.19)	26(63.41)		
	10,000–15,000 CNY	6(14.29)	13(31.71)		
	15,000–20,000 CNY	2(4.76)	1(2.44)		

### 3.2 Comparison of anxiety, psychological capital, experience, and support scores between the two groups of nurse second victims at baseline, immediately after intervention and 3 months post-intervention

At baseline (T1), no statistically significant differences were observed in GAD-7, PCQ-R, and SVEST scores (*p* = 0.596, *p* = 0.470, *p* = 0.435, respectively). Compared with the control group, the intervention group exhibited lower GAD-7 and SVEST scores and higher PCQ-R scores immediately after intervention (T2) and 3 months post-intervention (T3) (both *p* < 0.05 for GAD-7 and SVEST; all *p* < 0.01 for PCQ-R). See Table 3.

**Table 3:** Comparison of anxiety, psychological capital, second victim experience and support scores among nurse second victims at baseline, immediately after intervention, and 3 months post-intervention.

Group	*n*	GAD-7			PCQ-R			SVEST		
		T1	T2	T3	T1	T2	T3	T1	T2	T3
Intervention group	42	12.69 ± 4.57	8.74 ± 6.19	8.19 ± 5.61	86.40 ± 10.34	91.67 ± 13.96	93.48 ± 13.99	86.02 ± 11.50	78.38 ± 14.50	78.05 ± 11.91
Control group	41	13.22 ± 4.49	11.80 ± 5.15	11.02 ± 4.89	84.56 ± 12.70	83.17 ± 12.95	84.83 ± 12.98	87.88 ± 9.93	89.32 ± 13.62	87.17 ± 12.47
*t*		−0.532	−2.449	−2.453	0.726	2.872	2.917	−0.785	−3.540	−3.409
*p*		0.596	0.016	0.016	0.470	0.005	0.005	0.435	<0.001	0.001

### 3.3 Repeated-measures ANOVA for anxiety, psychological capital, second victim experience and support scores of nurse second victims in the two groups

The results revealed statistically significant main effects of time, between-group effect, and time-group interaction effects for GAD-7, PCQ-R, and SVEST scores among nurse second victims in both groups (*p* < 0.05), as shown in Table 4.

**Table 4:** Repeated-measures analysis of variance for anxiety, psychological capital, second victim experience, and support scores of nurse second victims in the two groups.

Dimension	Effect	*F*	df1	df2	*P*	partial η^2^
GAD-7	Between-group	4.490	1.000	81.000	0.037	0.053
GAD-7	Time	30.784	1.844	149.338	<0.001	0.275
GAD-7	Interaction	4.776	1.844	149.338	0.012	0.056
SVEST	Between-group	8.392	1.000	81.000	0.005	0.094
SVEST	Time	12.764	2.000	162.000	<0.001	0.136
SVEST	Interaction	14.402	2.000	162.000	<0.001	0.151
PCQ-R	Between-group	5.529	1.000	81.000	0.021	0.064
PCQ-R	Time	12.222	2.000	162.000	<0.001	0.131
PCQ-R	Interaction	13.375	2.000	162.000	<0.001	0.142

Mauchly’s test of sphericity indicated that for GAD-7, *W* = 0.915, χ^2^ = 7.096, df = 2, p = 0.029, with Greenhouse–Geisser ε = 0.922. Thus, Greenhouse–Geisser correction was applied for the time effect and group-by-time interaction effect. For PCQ-R:W = 0.996, χ^2^ = 0.281, df = 2, *p* = 0.869; for SVEST: *W* = 0.947, χ^2^ = 4.357, df = 2, *p* = 0.113. The sphericity assumption was satisfied for the latter two scales, partial η^2^ = partial eta-squared.

### 3.4 Within-group comparisons of anxiety, psychological capital, second victim experience and support scores for nurse second victims in the two groups across different time points

The results showed that in the intervention group, both GAD-7 and SVEST scores were lower after the intervention and 3 months post-intervention relative to baseline (all *p* < 0.05), while PCQ-R scores were higher compared with baseline (all *p* < 0.05). No statistically significant differences were observed between scores measured immediately after the intervention and those 3 months post-intervention (*p* > 0.05); In the control group, except for anxiety scores — which were lower after the intervention relative to baseline (*p* = 0.032, *p* < 0.001) — pairwise comparisons at all other time points showed no statistically significant differences (*p* > 0.05), as shown in Table 5.

**Table 5:** Within-group comparisons of anxiety, psychological capital, second victim experience and support scores among nurse second victims in the two groups at different time points.

Comparison groups	Scale	Control group			Intervention Group		
		Mean difference	Standard error	*P*	Mean difference	Standard error	*P*
T1–T2	GAD-7	1.415	0.529	0.032	3.952	0.752	<0.001
T1–T3		2.195	0.458	<0.001	4.500	0.591	<0.001
T2–T3		0.780	0.583	0.564	0.548	0.842	1.000
T1-T2	SVEST	−1.439	1.048	0.532	7.643	1.406	<0.001
T1–T3		0.707	0.935	1.000	7.976	1.172	<0.001
T2–T3		2.146	1.177	0.227	0.333	1.682	1.000
T1-T2	PCQ-R	1.390	0.891	0.380	−5.262	1.131	<0.001
T1–T3		−0.268	0.964	1.000	−7.071	1.120	<0.001
T2-T3		−1.659	1.049	0.365	−1.810	1.178	0.396

All *p*-values presented in the table are adjusted by the Bonferroni method for multiple comparisons. Mean differences were calculated as “earlier time point − later time point”.

## 4 Discussion

Nurses who become secondary victims commonly face dual pressures from career development and family obligations. The cumulative stress arising from multiple role demands can easily trigger persistent negative emotions that are difficult to regulate and release (Li et al., 2024). Furthermore, these nurses are exposed to stressors from multiple sources, including colleagues, patients, and public scrutiny, which may make them reluctant to engage in interpersonal communication and reduce social participation (Deng et al., 2025). Over time, secondary victims nurses often enter a cycle of negative self-evaluation: they continually magnify their mistakes and shortcomings related to adverse events and may even develop profound doubts regarding their professional competence. This accumulation of negative experiences elicits sustained negative affect such as anxiety, depression, and self-blame (Järvisalo et al., 2024). Adverse events constitute a chronic stressor for nurses affected by secondary victimization, further eroding self-efficacy and impairing their capacity to manage negative experiences. The present findings indicate that this intervention program effectively relieves anxiety symptoms, improved their psychological capital and support levels, and the intervention effects were sustained at the 3-month follow-up.

Related studies have shown (Kong, 2023) that multi-component positive psychology group interventions based on the PERMA model can promote nurses’ psychological well-being and mitigate negative affect, which aligns with the findings of this study. Built upon a trust-based group mutual-support framework, the present intervention created a safe, warm, and relaxing environment for secondary victim nurses. It focused on helping participants identify their internal positive strengths and foster positive interpersonal relationships, which gradually reduced their defensive and tense psychological states and thereby alleviating anxiety. The online component was delivered via “Nurse’s Mind” mini-program, which provided secondary victim nurses with stress monitoring, stress-relief games, and guidance for daily stress management practices including mindfulness-based stress reduction and meditation. These resources were designed to reduce their stress and anxiety and limit the emergence of negative emotions.

Psychological capital represents a vital positive psychological resource for individual growth, enabling them to mobilize internal psychological motivation (Gu et al., 2026). In this study, psychological capital scores in the intervention group increased significantly after the intervention and were higher than those in the control group (*p* < 0.05), consistent with earlier evidence (Yan and Zhang, 2021). This effect may be attributed to the PERMA-based multi-component positive psychology group intervention, which guided secondary victim nurses to interpret patient safety incidents rationally and optimistically, reframe their understanding of these events, and adopt positive coping mindsets to attenuate negative experiences. The intervention team conducted a series of positive psychology-oriented multi-component activities. These activities enhanced self-confidence by helping nurses recognize their professional value and sense of accomplishment as secondary victims and draw positive inspiration from peers’ achievements, further boosting psychological capital. The “Health Education Module” embedded in the mini-program supplied nurses who were secondary victims with evidence-based knowledge, mental health management guidance, and positive psychology and well-being education materials. This effectively helped individuals cultivate positive psychological resources and avoid psychological risks, supporting adaptive coping in adversity. Thus, this intervention program alleviates the negative experiences among secondary victim nurses while enhancing their psychological capital, fostering positive personal growth and strengthening resilience against future adversities.

For outcomes on the SVEST scale, the experience and support scores of secondary victim nurses in the intervention group were lower than those in the control group both immediately after the intervention and 3 months follow-up (*p* < 0.05). Since a lower SVEST score indicates higher perceived efficacy of available support resources, this finding suggests that the intervention program can effectively enhance support resource efficacy of secondary victim nurses, consistent with previous studies (Wang et al., 2022). The six-week hybrid online-offline group program established a peer support platform for these nurses, facilitating mutual communication, assistance, and trust-building, fostering positive support relationships among participants and thereby alleviating the helplessness following adverse events. Group sessions in the intervention program—such as the “Social Support Map”, “Emotional Management Training” and “On-Site Emotional First Aid Kit Techniques”— equipped secondary victim nurses with skills to rapidly identify personal and others’ emotions, offered positive psychological support resources, and strengthened psychological resilience. Additionally, the mini-program incorporated peer and psychological support modules, delivering online services such as “an anonymous peer discussion community”, “group mutual aid or online group counseling”, “rapid psychological assessment tools” and “anonymous one-on-one psychological counseling”. Through these multi-channel supports, the program enhanced nurses’ ability to cope with adverse events and further improved the perceived effectiveness of their support resources.

This study also has certain limitations. First, recruitment was limited to a single Class A tertiary comprehensive hospital in Shandong Province, which restricts the generalizability of the findings to other regions, hospitals tiers, or cultural contexts. Secondly, the follow-up duration was only 3 months; therefore, long-term sustained effects of the intervention could not be evaluated. Third, all outcome measures relied on self-reported questionnaires, rendering the data susceptible to social desirability bias, recall bias and common method variance. Future studies should incorporate objective indicators. Fourth, the intervention bundled multiple components, including emotional regulation, mindfulness, communication training and social support mapping. Without a dismantling study design, we cannot determine which individual component (s) produced the observed benefits, nor can we attribute effects exclusively to “positive psychology” itself. Fifth, we did not regularly monitor intervention fidelity or participants’ engagement with online modules (e.g., login frequency, content completion rates), so variability in the actual amount of dosage received may exist. Sixth, due to the nature of the intervention, participants and facilitators were unable to know the group assignments, which may introduce performance or detection bias. Future investigations may address these shortcomings through multi-center, large-scale randomized dismantling trials with extended follow-up (e.g., 6 or 12 months), objective outcome measures, fidelity monitoring and rigorous adherence documentation.

## 5 Conclusion

The findings of this study indicate that a multi-component positive psychology group intervention based on the PERMA model is feasible for nurses experiencing secondary victimization. Compared with the conventional control group, this intervention model effectively reduces anxiety symptoms and enhances psychological capital and perceived support efficacy among nurses affected by secondary victimization.

## Data Availability

The raw data supporting the conclusions of this article will be made available by the authors, without undue reservation.
